# High cardiac background activity limits ^99m^Tc-MIBI radioguided surgery in aortopulmonary window parathyroid adenomas

**DOI:** 10.1186/1471-2482-14-22

**Published:** 2014-04-23

**Authors:** Thomas Schwarzlmüller, Katrin Brauckhoff, Kristian Løvås, Martin Biermann, Michael Brauckhoff

**Affiliations:** 1 Department of Radiology, Centre for Nuclear Medicine/PET, Haukeland University Hospital, Jonas Liesvei 65, Bergen 5021, Norway; 2 Department of Surgery, Haukeland University Hospital, Bergen, Norway; 3 Department of Medicine/ Endocrinology, Haukeland University Hospital, Bergen, Norway; 4 Department of Clinical Science, University of Bergen, Pb 7804, Bergen 5020, Norway; 5 Department of Clinical Medicine, University of Bergen, Pb 7804, Bergen 5020, Norway

**Keywords:** ^99m^Tc-MIBI scintigraphy, Aortopulmonary window parathyroid adenoma, Radioguided surgery, Primary hyperparathyroidism

## Abstract

**Background:**

Radioguided surgery using 99m-Technetium-methoxyisobutylisonitrile (^99m^Tc-MIBI) has been recommended for the surgical treatment of mediastinal parathyroid adenomas. However, high myocardial ^99m^Tc-MIBI uptake may limit the feasibility of radioguided surgery in aortopulmonary window parathyroid adenoma.

**Case presentation:**

Two female patients aged 72 (#1) and 79 years (#2) with primary hyperparathyroidism caused by parathyroid adenomas in the aortopulmonary window were operated by transsternal radioguided surgery. After intravenous injection of 370 MBq ^99m^Tc-MIBI at start of surgery, the maximum radioactive intensity (as counts per second) was measured over several body regions using a gamma probe before and after removal of the parathyroid adenoma. Relative radioactivity was calculated in relation to the measured *ex vivo* radioactivity of the adenoma, which was set to 1.0.

Both patients were cured by uneventful removal of aortopulmonary window parathyroid adenomas of 4400 (#1) and 985 mg (#2). Biochemical cure was documented by intraoperative measurement of parathyroid hormone as well as follow-up examination. *Ex vivo* radioactivity over the parathyroid adenomas was 196 (#1) and 855 counts per second (#2). Before parathyroidectomy, relative radioactivity over the aortopulmonary window versus the heart was found at 1.3 versus 2.6 (#1) and 1.8 versus 4.8 (#2). After removal of the adenomas, radioactivity within the aortopulmonary window was only slightly reduced.

**Conclusion:**

High myocardial uptake of ^99m^Tc-MIBI limits the feasibility of radioguided surgery in aortopulmonary parathyroid adenoma.

## Background

Radioguided surgery (RGS) using the radioactive tracer 99m-Technetium-methoxyisobutylisonitrile (^99m^Tc-MIBI) has been recommended for treating patients with primary hyperparathyroidism (PHPT), in particular in recurrent disease and atypical localization of the parathyroid glands (e.g. in the mediastinum) [1-4]. Potential benefits of RGS are (a) better identification of pathological parathyroid tissue by intraoperative detection of regions with highest tracer uptake and (b) control of cure by comparison of the *ex vivo* radioactivity over the removed tissue and *in vivo* activity within the resection area [5-7].

The main prerogative of this method is the selective (or higher) uptake of the tracer in parathyroid tissue when compared to the surrounding tissue [8-10]. ^99m^Tc-MIBI, a tracer that was originally developed for myocardial scintigraphy, has however physiological uptake in the myocardium as well as uptake in many thyroid nodules including thyroid cancer [9,11]. For reliable discrimination in cervical PHPT surgery, a ratio between thyroid tissue and parathyroid adenoma of at least 1:1.3-1.5 has been suggested [4,12,13]. After removal of the parathyroid adenoma, an *ex vivo* radioactivity counting rate over the adenoma of more than 20% compared to the thyroid background [4,13,14] and the surgical bed is considered to be a credible criterion for successful excision [13].

Several studies have been published concerning a benefit for RGS in intraoperative identification of mediastinal parathyroid adenomas [5,15-17]. The majority of mediastinal parathyroid adenomas are localized in the thymus, the anterior superior mediastinum, or the tracheo-oesophageal groove in the upper mediastinum [6,16,18].

In less than 1 of 1,000 patients, however, parathyroid adenomas can be found in the aortopulmonary window (APW), which is localized deeper and more posterior in the mediastinum between the aorta and the left pulmonary artery, in the region of the tracheal bifurcation and near to the heart [15,19,20]. In APW parathyroid adenomas RGS has been tested in only a few cases [20]. In contrast to the neck, no standard protocol has so far been established for RGS in APW parathyroid adenomas [2]. In particular, the high myocardial MIBI uptake may be a limiting factor.

The present study was undertaken to examine the feasibility of RGS for APW parathyroid adenoma. Main aim of the study was to evaluate the usefulness of this method regarding (a) intraoperative localization of the adenoma and (b) control of the surgical result (cure of disease).

## Case presentation

### Patients

Two female patients, aged 72 (patient #1) and 79 years (patient #2), with PHPT due to an APW parathyroid adenoma were included in this study. Both patients presented with typical PHPT reflected by ionized serum calcium levels of 1.73 (#1) and 1.61 mmol/L (#2), total serum calcium levels of 3.08 (#1) and 3.04 mmol/L (#2), serum phosphate of 0.75 (#1) and 0.64 mmol/L (#2), and elevated parathyroid hormone (PTH) of 85.9 (#1) and 90.2 pmol/L (#2) (normal upper value below 6.8), respectively.

**Figure 1:**
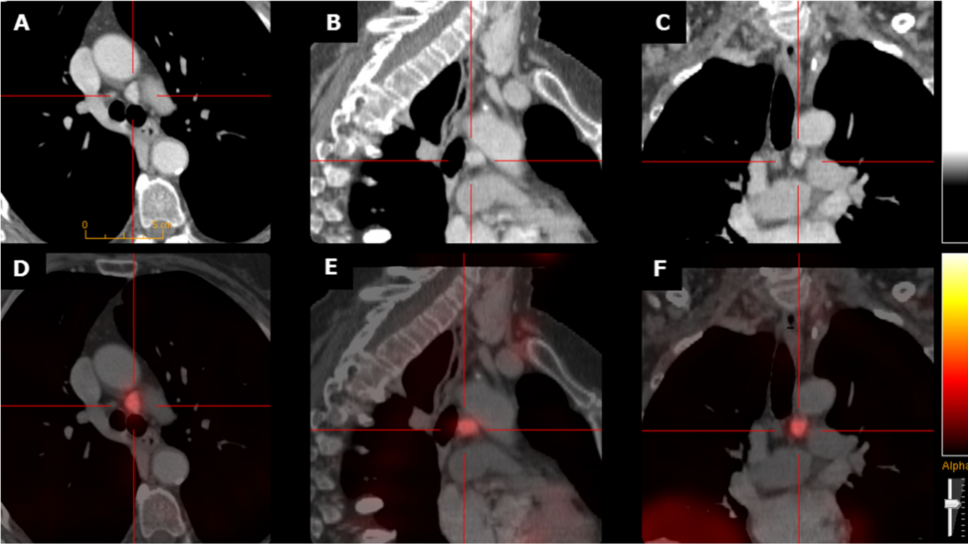
**Aortopulmonary window parathyroid adenoma in patient #2: contrast**-**enhanced CT (CE**-**CT; panels A-C) and CE-CT co**-**registered with **^**99m**^**Tc-MIBI SPECT (panels D-F) in axial (A, D), sagittal (B, E), and coronal (C, F) orientation.**

Single photon emission computed tomography/X-ray computed tomography (SPECT/CT) with ^99m^Tc-MIBI and contrast-enhanced computed tomography (CECT) [21] identified ectopic parathyroid adenomas in the APW in both patients (axial diameter 35 (#1) and 12 mm (#2), respectively) (Figure 1). Cervical ultrasound was negative.

Clinically, the first patient (#1) had moderate osteopenia but no other typical PHPT associated symptoms. The second patient (#2), an obese type-2 diabetic with hypertension and atrial fibrillation, presented with confusion and malaise combined with dehydration and vomiting.

Based on the imaging findings, both patients were scheduled for transsternal surgery without cervical exploration. Both patients gave their informed consent for this particular clinical study.

### Radioguided surgery and radioactivity measurement

At the start of surgery, 370 MBq ^99m^Tc-MIBI were injected intravenously. Surgery was performed using intraoperative neuromonitoring of the recurrent laryngeal nerve and intraoperative measurement of PTH. After sternotomy, the space between the inferior margin of the aortic arch and the left pulmonary artery was carefully dissected. The ligament of Botalli, the left recurrent nerve and the vagus nerves were identified. 60, 90, and 120 minutes after MIBI-injection, radioactivity was measured (in counts per second, cps) over several areas using a gamma probe, equipped with internal tungsten shielding and a reusable external collimated detector (neo2000® GDS, Neoprobe Corp., Dublin/OH, USA; 14 mm probe, spatial resolution 15 mm, angular resolution 25°). As testing areas were selected: 1) the APW, 2) the left ventricle, 3) the left thyroid lobe, 4) the right lung, and 5) the lower abdomen. Radioactivity was measured within an area of about 30 mm in diameter over these regions and the mean value was used as counted radioactivity over each region.

After removal of the adenoma (120 minutes after MIBI-injection), the *ex vivo* activity of the parathyroid adenoma was measured.

For intraoperative PTH measurement, blood samples were taken before surgery, shortly before (pre-excision), and 5, 10, and 20 minutes after removal of the parathyroid adenoma.

The removed specimens were examined histologically.

Postoperatively, blood samples for ionized and total serum calcium and plasma PTH were taken each day until discharge from hospital, thereafter 3 months after surgery. Furthermore, vocal cord motility was assessed postoperatively by direct laryngoscopy.

## Results

### Surgical results

**Figure 2:**
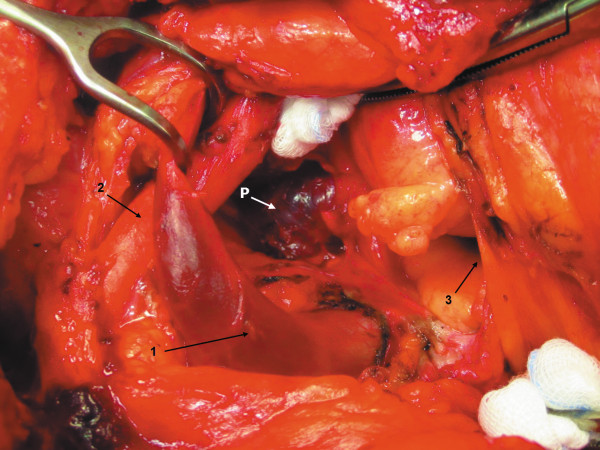
**Aortopulmonary window adenoma before resection (patient #1).** Aortic arch under the pledget. P = Parathyroid adenoma, 1 = Left brachiocephalic vein and vena cava, 2 = Brachiocephalic trunc, 3 = Opened pericardium.

**Figure 3:**
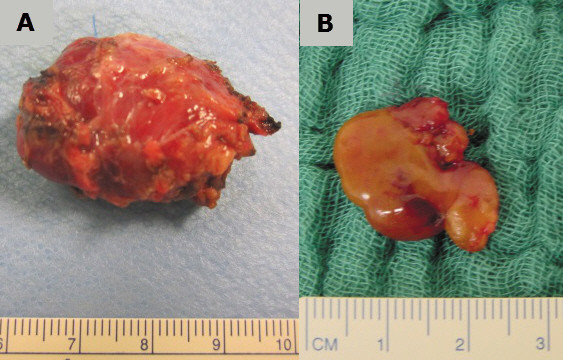
**Removed aortopulmonary parathyroid adenomas in patient #1 (panel A) and patient #2 (B).**

In both patients, surgery was uneventful. The parathyroid adenomas were identified visually in the APW within the expected position (Figure 2). In the first patient (#1), the adenoma presented as a cystic tumour of about 35 mm in diameter; in the second patient (#2), the adenoma was solid with a diameter of about 15 mm (Figure 3). After removal of the adenomas (weight: 4,400 (#1) and 985 mg (#2), respectively), PTH decreased within 10 minutes into the normal range: 89.3 (#1) and 59.2 pmol/L (#2) before excision versus 5.7 (#1) and 2.5 pmol/L (#2) ten minutes after resection.

Serum calcium and PTH were in the normal range three months after surgery in both patients. No permanent recurrent nerve palsy was observed. Patient #2 showed a marked clinical improvement within a few days after surgery.

Histological examination revealed classical parathyroid adenoma, in the first patient (#1) with cystic degeneration.

### Radioactivity measurements

The *ex vivo* count rate over the removed parathyroid adenomas 120 minutes after MIBI-injection were 196 cps in the cystic adenoma (#1) and 855 cps in the solid adenoma (#2). Compared to these *ex vivo* activities, all other counted activities were calculated as relative radioactivity (RRA) compared to the *ex vivo* count rates.

**Figure 4:**
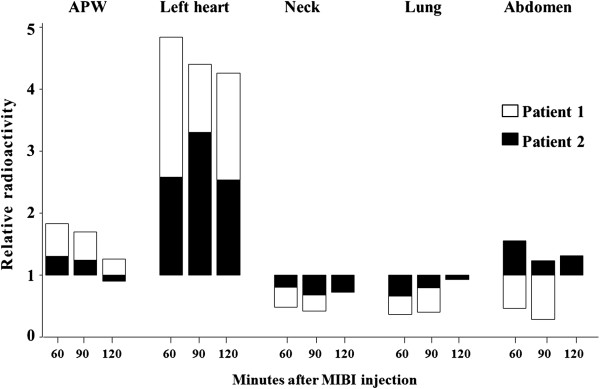
**Relative radioactivity (RAA) 60, 90, and 120 minutes after radioactive tracer injection over different tissues in both patients with aortopulmonary window parathyroid adenoma (Parathyroid adenoma ex vivo RAA = 1.0).**

The relative radioactivities 60, 90 and 120 minutes after MIBI administration differed in all the tested regions (Figure 4).

Before removal of the parathyroid adenomas, the relative radioactivities over the APW were above 1.5 in both patients. No differences in counted radioactivity were found over the APW within the tested area of about 30 mm in diameter. However, the direction of the collimated gamma probe had a marked effect on the measured count rates, which were highest when the probe was angled towards the heart. For measurement of the RRA over the APW region, the gamma probe was therefore always pointed in a straight posterior direction. After excision of the parathyroid adenoma, the relative radioactivities in the APW decreased to 1.16 (#1) and 0.89 (#2), respectively, while relative radioactivities over the left ventricle, about 2.5–3 cm distant from the tested APW region, ranged between 2.0 and 5.0.

In contrast to the APW and the left heart, the relative radioactivities over the left neck and the right lung were always clearly below 1.0. Over time, one patient (#1) had slightly increasing relative radioactivities over the abdomen, which probably was due to tracer accumulation in the urinary bladder as this patient was not catheterized (Figure 4).

### Discussion

In this exploratory study, we examined the feasibility of RGS in two patients with APW parathyroid adenoma using a transsternal approach. As expected from the physiological tracer distribution, very high relative radioactivities were found over the heart on all measurements whereas the relative radioactivities over the lung and the neck were clearly below 1.0. Within the APW, no differences in count rates were detected within an area of 30 mm in diameter, approximately twice the diameter of the probe face. In this area, the relative radioactivities before removal of the parathyroid adenomas were always clearly above 1.0. Concerning intraoperative localization (guidance) of the adenoma, however, RGS had to be assessed as not very helpful. Removal of the parathyroid adenoma led to a slight reduction of radioactivity within this area in both patients. The relative radioactivities, however, were still around 1.0, and higher when compared to the relative radioactivities over the neck and the lung.

### Background activity as the key for RGS

During the last two decades, preoperative multimodality imaging, minimal invasive parathyroidectomy, intraoperative PTH measurement, and RGS have had a profound effect on parathyroid surgery and become routine at many endocrine surgical centres [15,18,19]. RGS using ^99m^Tc-MIBI has been proposed to be particularly useful in patients with ectopic parathyroid adenomas [2,5,12,20,22,23]. Numerous application protocols regarding RGS have been reported, with injected radioactivity ranging from 37 to 740 MBq ^99m^Tc-MIBI. Concerning the time interval between application and scanning, protocols are rather similar with tracer injection around the start of surgery, which usually results in an interval of about 30 to 90 minutes [1-4,22]. Administration of 370 MBq is probably most common [2,4]. This protocol seems to be the best compromise regarding detection accuracy and radiation protection of the personnel.

^99m^Tc-MIBI was originally developed for myocardial scintigraphy [24]. The lipophilic tracer is taken up and bound to the mitochondria of viable myocardium as well as other hypermetabolic mitochondria-rich tissues. Thus it is more avidly taken up by parathyroid adenomas and hyperplastic parathyroid tissue than by normal parathyroid glands [9,11]. Uptake of ^99m^Tc-MIBI is, however, not specific to parathyroid tissue. Consequently, for detection of abnormal hyperfunctioning parathyroid tissue, different uptake kinetics between adenoma or hyperplastic glands and the surrounding tissue are required. Most of the protocols in the neck are using a 20% cut-off. Based on that criterion, surgery is considered successful when radioactivity of the removed specimen is more than 20% higher than that of the surrounding (thyroid) tissue [4,13,14].

### RGS for localization of APW adenoma

This study shows that RGS for localization of APW parathyroid adenoma is limited by high myocardial background activity. Due to the short distance between the heart and the APW region, the RRA within the APW region remained relatively high even after removal of the parathyroid adenoma. The count rates substantially depended on probe direction. Therefore, in our experience, RGS is not very helpful concerning intraoperative localization of APW parathyroid adenomas when performing surgery via a transsternal approach.

Some surgeons, however, prefer left thoracotomy for removing APW parathyroid adenoma [19,25-28], which might lead to an improved discrimination due to a different position of the gamma probe. Furthermore, during the last decade, an increasing number of reports regarding minimal-invasive surgery of mediastinal parathyroid adenoma have been published [29,30]. Even though most of these adenomas were localized in the upper and anterior mediastinum, the feasibility of minimal-invasive surgery in APW parathyroid adenoma using a robot-assisted approach has been demonstrated [31,32]. None of these robot-assisted operations, however, focused on RGS. Important enough, RGS for APW parathyroid adenoma surgery would probably require special equipment since the usual commercial gamma probes are not designed for this approach. Thus, the feasibility of RGS in APW parathyroid adenoma using robot-assisted surgery remains to be demonstrated.

### Testing of cure

After removal of the parathyroid adenomas, a clear RRA reduction within the APW region was found. In addition, the activity over the removed adenomas was much higher when compared to other reference areas (neck, lung). On the other hand, however, the RRA over the APW was still around 1.0 after removal of the adenomas.

Regardless the extent of RRA decrease, decreasing activity does not give evidence of biochemical cure at all since other hyperfunctioning glands (e.g. in the neck) cannot be excluded. Since intraoperative PTH measurement showed normalization within 10 minutes in both patients, no further surgery or exploration was necessary in the two patients.

## Conclusion

Lacking a clear benefit of RGS in transsternal surgery of APW parathyroid adenoma and taken the potential side effects (e.g. radioactive exposure of the patient and the personnel) into consideration, RGS cannot be recommended in those patients. Whether another approach or another protocol (e.g. a longer interval between tracer injection and measurement) would improve the results remains to be clarified.

### Consent

Written informed consent was obtained from the patients for publication of this Case report and any accompanying images. A copy of the written consent is available for review by the Editor of this journal.

## Abbreviations

APW: Aortopulmonary window; CECT: Contrast enhanced computed tomography (X-ray); MBq: Megabecquerel; Tc-MIBI: 99 m-Technetium-methoxyisobutylisonitrile; PHPT: Primary hyperparathyroidism; PTH: Parathyroid hormone; RGS: Radioguided surgery; RRA: Relative radioactivity; SPECT/CT: Single photon emission computed tomography/X-ray computed tomography.

## Competing interests

The authors declare that they have no competing interests.

## Authors’ contributions

TS: assisted with the surgical procedures, conducted a comprehensive literature search, analysis and interpretation of data and drafting of the manuscript. KB: performed, assisted with the surgical procedures and the research, clinical evaluation. KL, MB: monitored the drafting process, assisted with the research, literature search and critical revision of the manuscript. MB (last author): clinical evaluation, performed the surgical procedures, analysis and interpretation of data, reviewed the manuscript and gave the final approval for publication. All authors read and approved the final manuscript.

## Pre-publication history

The pre-publication history for this paper can be accessed here:

http://www.biomedcentral.com/1471-2482/14/22/prepub
